# The effect of LTP- and LTD-like visual stimulation on modulation of human orientation discrimination

**DOI:** 10.1038/s41598-018-34276-z

**Published:** 2018-11-01

**Authors:** Andreas Marzoll, Tan Saygi, Hubert R. Dinse

**Affiliations:** 1 Neural Plasticity Lab, Institute for Neuroinformatics, Ruhr-University Bochum, Bochum, Germany; 2 Department of Neurology, BG University Hospital Bergmannsheil, Ruhr-University Bochum, Bochum, Germany

## Abstract

Studies showing that repetitive visual stimulation protocols alter perception and induce cortical reorganization, as well-reported for the tactile domain, have been sparse. In this study, we investigated how “long-term potentiation [LTP]-like” and “long-term depression [LTD]-like” repetitive visual stimulation affects orientation discrimination ability in human observers. LTP-like stimulation with features most closely resembling the stimuli used during behavioral assessment evoked the largest improvement, while the effects were smaller in protocols that differed in shape or orientation features. This gradient suggests lower learning specificity than classical perceptual learning experiments, possibly because of an interplay of task- and feature-based factors. All modulatory effects of repetitive stimulation were superimposed on top of spontaneous task learning. Moreover, blockwise analysis revealed that LTP-like stimulation, in contrast to LTD-like or sham stimulation, prevented a loss of practice-related gain of orientation discrimination thresholds. This observation highlights a critical role of LTP-like stimulation for consolidation, typically observed during sleep.

## Introduction

A large body of research from the last decades has shown that perceptual abilities are modifiable through training and practicing^1–3^. While the classical method to induce perceptual learning consists of deliberately practicing a perceptual task, novel approaches were discovered in recent years that could induce comparable perceptual gains, including learning by neurofeedback^4^, mental imagery^5,6^, repetitive transcranial magnetic stimulation^7–9^, direct-current stimulation^10^, random-noise stimulation^11–13^, or task-irrelevant perceptual learning^14^. Furthermore, combining factors known to facilitate perceptual learning can increase its efficacy^15,16^.

The approach explored here was to drive perceptual improvement by repetitive sensory stimulation. Our strategy followed the idea of electrical stimulation protocols developed to directly induce frequency-dependent synaptic plasticity in *in vivo* and *in vitro* cellular experiments, such as long-term potentiation (LTP) or long-term depression (LTD)^17–19^. Indeed, tactile stimulation of the index finger using a high-frequency intermittent “LTP-like” tactile stimulation for only 20 minutes improved tactile acuity selectively on the stimulated finger, while continuous low-frequency “LTD-like” stimulation impaired acuity^20^. These perceptual changes were paralleled by an expansion of the cortical territory representing the stimulated fingers in the primary and secondary somatosensory cortex^21^. Most interestingly, in the S1 region the degree of map reorganization correlated with the individual perceptual gain in tactile acuity, indicating a causal relationship between plastic map changes and perceptual learning effects^21^. These forms of plasticity, induced by repetitive sensory stimulation, were shown to depend on activation of the N-methyl-D-aspartate (NMDA) receptor, as pharmacological blocking of NMDA receptors through a single dose of memantine abolished plastic changes both at the behavioral and the cortical level^22^. Altogether, the available data from the somatosensory system indicate that repetitive sensory stimulation is a powerful tool to induce processes of synaptic plasticity at the neural sites representing the stimulated skin portion, thereby leading to wide-range remodeling of tactile perception and cortical processing^23–28^.

As to modality-specific aspects of RSS-induced plasticity in the tactile and visual system, both systems are characterized by input channels that differ in temporal transfer properties. In touch, a variety of mechanoreceptors differing in transfer properties channel incoming information to the brain^29^. Most likely, FA-I (fast-adapting type I) Meissner endings sensitive to dynamic skin deformation of relatively high frequency are involved in transmitting the 20 Hz stimulation used during repetitive tactile stimulation. In addition, the FA-II (fast-adapting type II) Pacini endings, which are highly sensitive to mechanical transients and high-frequency vibrations might contribute as well. In vision, three main parallel input streams arise from different types of ganglion cells, the magnocellular, parvocellular and koniocellular pathways, that project to the central visual system^30^. During LTP-like 20 Hz visual stimulation, most likely the magnocellular pathway will be involved.

While the above considerations relate to peripheral input properties, aspects reflecting cortical response properties may be more important. Steady-state evoked potentials (EPs) are recorded using repetitive stimuli in a range between 5 and 50 Hz, and are analyzed using a frequency domain analysis. In the visual system, the amplitudes of steady-state EPs are largest close to 10 and 18 Hz for unpatterned flash stimuli and at somewhat lower frequencies for patterned stimuli^31^. In the somatosensory system, our results and those of Snyder^32^ showed the greatest S/N ratio of the S-SEP amplitude at a frequency range of 21 to 26 Hz. The frequency of 20 Hz used during repetitive tactile stimulation fits the preferred frequency range reported for SI cortex. On the other hand, the visual stimulation uses a 20 Hz frequency as well, but in addition employs a 50 ms presentation time for the visual stimuli resulting in an additional 10 Hz component. Accordingly, the preferred frequency range reported for visual cortex is matched as well. So despite major differences in receptors properties and pathways, the prerequisites for a faithful transmission of the temporal pattern used in repetitive stimulation are given in both the tactile and visual modality.

For the visual modality, little is known about the effectiveness of repetitive visual stimulation approaches and their association to LTP/LTD. Frenkel *et al*.^33^ increased the amplitude of visual-evoked potentials (VEPs) recorded in the mouse V1 by stimulation with contrast-reversing gratings at 1 Hz. This effect became apparent over the course of several recording days and was found to depend on NMDA receptor function and unimpeded trafficking of the α-amino-3-hydroxy-5-methyl-4-isoxazolepropionic acid (AMPA) receptor. Only 2 min of 9-Hz stimulation with checkerboard stimuli led to an increase of VEP amplitude in rats that lasted up to 5 h and was located in the V1b area^34^.

LTP-/LTD-like visual stimulation approaches have been used to modulate the detection of relevant and irrelevant stimuli in a change detection task^35^, and in a face recognition task^36^. While these studies addressed the question of whether this approach is suitable for the investigation of higher order cognitive functions, little is known about the effectiveness of LTP-/LTD-like visual stimulation in altering low-level perceptual abilities as was the case in the tactile domain, where most studies assessed changes of tactile spatial discrimination as a marker of plastic changes^20–22^.

Indeed, recent studies have shown that LTP-like stimuli alter processing in the human visual cortex. For example, 2 min of visual high-frequency stimulation lead to specific changes in the early visual cortex. After stimulation with a checkerboard stimulus, VEPs showed increased amplitude of the N1b component that was strongest in the hemisphere contralateral to the stimulated visual hemifield^37^. A functional magnetic resonance imaging (fMRI) study using the same paradigm demonstrated a bilateral increase in blood oxygen level-dependent contrast imaging signal after stimulation that was confined to extrastriate areas when the stimulated hemifield was presented with the checkerboard stimulus^38^. High-frequency stimulation with gratings showed that the enhancing effects on VEP amplitude are specific for stimulus parameters such as spatial frequency^39^, and orientation^40^. However, there is a dearth of behavioral assessments for this type of repetitive sensory stimulation.

A prominent feature of the primary visual cortex is the presence of neurons showing specificity for the orientation of a stimulus. It is well-documented that human participants can substantially improve their fine orientation discrimination after several days of practicing^41,42^. In the present study, we asked whether 40 min of LTP- and LTD-like visual stimulation without any task training are sufficient to improve or impair performance in a fine orientation discrimination task, as described by Schoups, Vogels, & Orban^41^. In this task, the participants were asked to report the deviation of an oriented noise field from a not-shown 45° orientation either as clockwise or anticlockwise. In contrast to Schoups *et al*., we used this task not for training over many days, but only for assessment of discrimination thresholds before and after application of LTP- and LTD-like visual stimulation within two sessions run on one day. According to the data of Schoups *et al*., practice-induced improvement within a daily session was very small, suggesting the need for an overnight consolidation period. Notably, testing different time periods between retesting revealed that improvement of performance requires a latent phase, such as a night’s rest^41^. Considering these findings, we hypothesized that LTP-/LTD-like stimulation should be delivered between two sessions on the same day to evoke measurable modulation of performance compared with a non-stimulation condition.

As a particular property of this type of perceptual learning, practice-induced improvements in discrimination thresholds were shown to depend on the trained orientation^41^. Thus, in further experiments we explored spatial and orientation specificity of the visual stimuli used for LTP-like stimulation. We hypothesized that LTP-like visual stimulation would improve discrimination performance, while LTD-like stimulation would impair it. Moreover, we expected a comparable degree of stimulus specificity for stimulation-based learning. While our data showed a significant protocol-dependent modulation of discrimination performance supporting the effectiveness of repetitive stimulation in the visual domain, we found little dependence on the orientation used for induction, which might indicate different mechanisms underlying stimulation and practice-induced perceptual learning.

## Results

### Repetitive visual stimulation alters orientation discrimination

**Figure 1:**
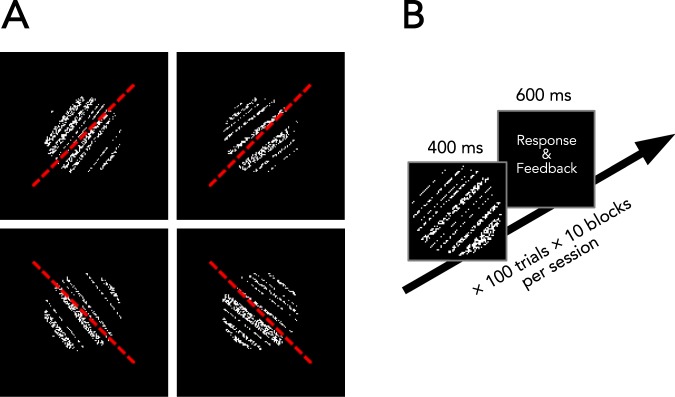
Orientation discrimination task. The observers judged whether a noise field stimulus with superimposed dark stripes differed either clockwise or counter-clockwise from an unseen target orientation. (**A**) Sample stimuli for both right and left oblique reference orientations indicated by a dashed red line (not actually shown to the subjects). (**B**) Time course of a trial, during which auditory feedback was provided in response to a wrong answer or a missed trial. One block consisted of 100 trials, for which individual JNDs were calculated. Per session (pre/post), 10 blocks were performed.

**Figure 2:**
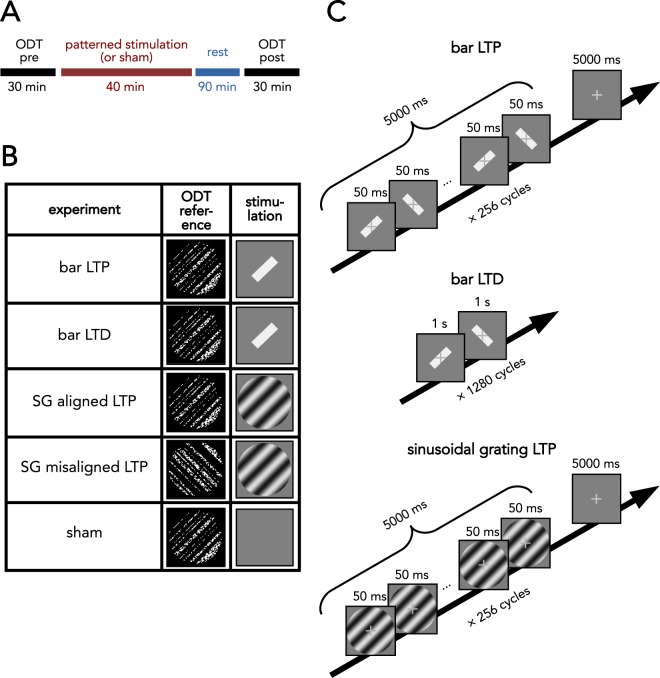
Experimental design and stimulation protocols. (**A**) General time course of an experiment. (**B**) Five experiments were conducted. Between pre- and post-sessions of an orientation discrimination task (ODT), different interventions were applied to the subjects. “Bar LTP”: a rectangular stimulus which flickered intermittently at a high frequency between the right and left oblique orientation was used during visual stimulation. “Bar LTD”: a rectangular stimulus which flickered continuously at a low frequency between the right and left oblique orientation was used during visual stimulation. “SG-aligned LTP”: sinusoidal grating (SG) was used for stimulation which had the same orientation (right oblique) as the target orientation during the orientation discrimination task. “SG-misaligned LTP”: the same stimulation was used as in the above condition, but the orientation used during the orientation discrimination task was rotated by 90°. “Sham”: no stimulus except for the fixation cross was shown during visual stimulation. (**C**) For LTP-like stimulation, one stimulation cycle lasted for 10 s, during which one of two stimulus pairs flickered at 20 Hz, for the first 5 s. During the latter 5 s, only the fixation cross was visible. This cycle was repeated 256 times. “bar LTP”: stimulus pair used in the bar condition. Two elongated rectangular stimuli flickered between the left and right oblique orientations (±45°). “bar LTD”: LTD-like stimulation used the same bar-like stimuli as in the LTP-like protocol; both oblique orientations were changed continuously at a frequency of 1 Hz. Thus, one stimulation cycle took 2 s and was repeated 1280 times, matching the total duration of the other stimulation protocols. “SG LTP”: stimulus pair used in the SG aligned LTP and SG misaligned LTP experiments. The SG changed spatial phase by 180° within a stimulation cycle. Between cycles, the phase was randomized.

We measured orientation discrimination thresholds (just-noticeable difference, JND) as a marker of visual perceptual performance. The subjects had to report whether an oriented noise field, as adopted from Schoups, Vogels, & Orban^41^, deviated clockwise or counter-clockwise from an unseen right or left oblique target orientation (Fig. 1). JNDs were measured prior to and after approximately 40 min of repetitive visual stimulation (Fig. 2A). We used stimulation protocols consisting of different timing and of different spatial features (Fig. 2).

We calculated a permutated mixed-model ANOVA to elucidate the effects of different interventions on orientation discrimination ability, as JNDs were generally not normally distributed and homoscedastic, with a notable negative skew (see Material & Methods). There was a significant within-subject effect of session (p < 0.001) and a significant between-subjects effect of stimulation type (p = 0.049), but no significant interaction between the two (p = 0.18).

**Figure 3:**
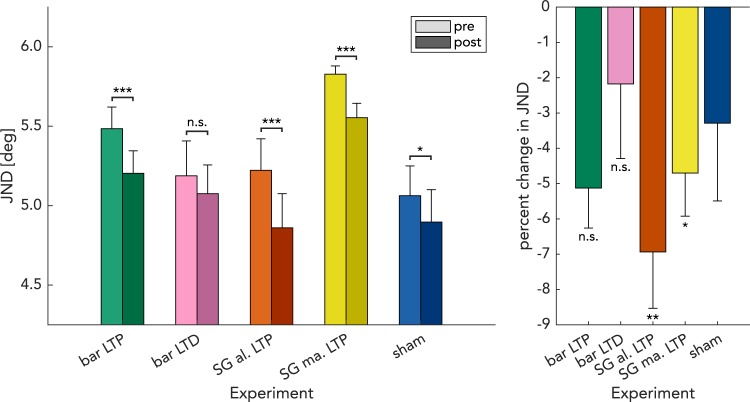
Visual stimulation improves orientation discrimination performance. Left: Bar diagrams show the change in mean JNDs after visual stimulation for each condition. Significance of Wilcoxon signed-ranks tests for each group is indicated in the diagram. Significance levels of post-hoc Wilcoxon tests are indicated here. Right: Percent change of JNDs after visual stimulation. Significance levels of bootstrapped t-tests that indicate significant differences from pre-post differences in the sham experiment are indicated here. Green: bar LTP. Purple: bar LTD. Red: SG-aligned LTP. Yellow: SG-misaligned LTP. Blue: sham. Error bars indicate standard error of the mean.

In the first experiment, we tested the effect of an LTP-like visual stimulation using an oriented bar-like stimulus^35^, which flickered intermittently between the two oblique orientations, at a rate of 20 Hz (Fig. 2B,C, “bar LTP”). In the tactile domain, LTP-like stimulation typically induces an improvement in tactile acuity^20–22^. Therefore, we expected that the orientation discrimination thresholds would be lower after stimulation. Indeed, orientation discrimination thresholds for the right oblique target orientation reduced from 5.48° ± 0.14° JND prior to stimulation to 5.20° ± 0.14° JND (mean ± standard error of the mean) after stimulation (Fig. 3, “bar LTP”). A Wilcoxon signed-ranks test indicated significant improvement (Z = 3.54; p < 0.001) of 5.1% (n = 25). Overall, 24.0% of the subjects (6 out of 25) did not show an improved performance, as indicated by a pre-post JND-difference larger than or equal to 0.

In the second experiment, we used the LTD-like visual stimulation consisting of continuous low-frequency presentation of the same bar stimulus as in the LTP-like protocol (Fig. 2B,C, “bar LTD”). In the tactile domain, LTD-like stimulation typically induces impaired tactile acuity^20^. We therefore expected that orientation discrimination thresholds should be higher after stimulation. Instead, JNDs only marginally changed from 5.19° ± 0.22 to 5.08° ± 0.18 after stimulation (Fig. 3, “bar LTD”), reflecting a nonsignificant (Z = 1.03, p = 0.30) difference of 1.4% (n = 16). In this group, 43.8% of the participants (7 out of 16) showed impaired performance after stimulation.

In two further experiments, we addressed the feature specificity of these effects. Using an LTP-like protocol, we investigated whether the efficacy of stimulation depends on the shape and pattern of the oriented stimuli. Thus, in the third experiment, we presented a sinusoidal grating (SG) with an orientation aligned to the reference orientation of the discrimination task, and of similar size (Fig. 2B,C, “SG aligned LTP”). In the fourth experiment, the orientation of the gratings was rotated by 90° (Fig. 2B,C, “SG misaligned LTP”). We expected greater learning to occur upon stimulation with the aligned grating than with the misaligned one. For the aligned grating, we measured an orientation discrimination threshold improvement from 5.25° ± 0.19° JND to 4.89 ± 0.20° JND (Fig. 3, “SG aligned LTP”) (Z = 4.02; p < 0.001), reflecting a significant improvement of 7.3% (n = 25). Only 16.0% of all subjects (4 out of 25) did not show a performance improvement. In contrast, for the misaligned grating, we found that subjects improved their threshold from 5.76° ± 0.08° JND to 5.50° ± 0.11° JND (Fig. 3, “SG misaligned LTP”), (Z = 3.98; p < 0.001), reflecting a significant improvement of 4.6% (n = 23). Among all participants, 13.0% (3 out of 23) did not show an improvement. Please note that in this experiment baseline performance is notably higher than in the others, while SEM is lower. Even though the reference orientation in the task is different, prior results have shown that orientation discrimination performance at the oblique orientations is comparable^43^. We assume that the different performance here is due to chance (lower SEMs can be explained by the fact that more subjects were approaching the maximum orientation deviation of 7°).

In the fifth experiment, the subjects received a sham stimulation serving as control for the other conditions (Fig. 2B, “sham”). The subjects were exposed to a stimulation protocol of the same duration as those in the other experiments, but no stimuli were shown except for a fixation cross. We found a significant difference in this setup (Fig. 3, “sham”), pre JND: 5.09° ± 0.18°; post JND: 4.93° ± 0.20°; ΔJND = 3.2%; Z = 1.89; p = 0.048; n = 25. In this condition, 43.8% of the subjects (6 out of 25) showed no improvement in performance. Given these data, we cannot postulate a lack of improvement in this experiment to contrast the other effects with. Therefore, we performed further tests—employing a bootstrap method (see below)—that directly compare behavioral changes with those found under control conditions.

To further quantify the effects seen in the different conditions, we calculated the effect sizes for the four stimulation experiments with respect to the effects obtained in the sham experiment as the standardized mean difference (SMD). For the “SG-aligned LTP” experiment, we found a medium effect size of SMD = −0.35. For both “SG misaligned LTP” and “bar LTP” experiments, smaller effects of SMD = −0.20 and −0.23, respectively, were found. In the LTD-like experiment, we found a small effect size of SMD = 0.08.

### Bootstrap

**Figure 4:**
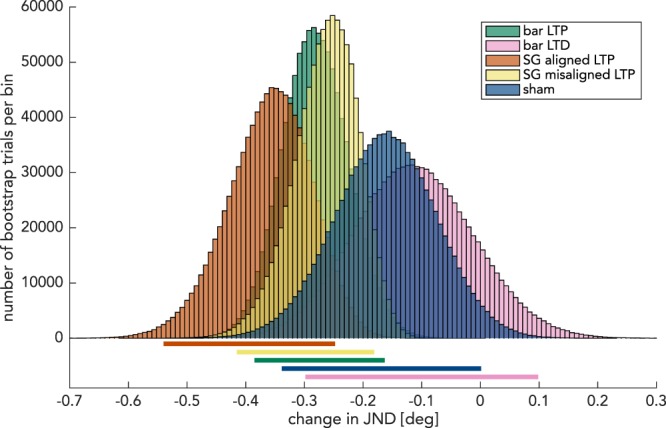
Bootstrap analysis. Histogram of the bootstrapped change in JND for every experiment. 95% confidence intervals using the “bias corrected and accelerated,” method which correspond to these distributions are shown below the horizontal axis. For each experiment, 1,000,000 bootstrap simulations were computed. Green: bar LTP. Purple: bar LTD. Red: SG-aligned LTP. Yellow: SG-misaligned LTP. Blue: sham. Bin width: 0.008°.

As an additional approach to demonstrate that the effects of LTP/LTD-like stimulation were different from the changes seen in the sham experiment, we conducted a bootstrapped version of a paired samples t-test for these four cohorts against the null-hypothesis that the change in JND was no different from that recorded in the sham experiment (−0.17° difference in JND after stimulation). This analysis revealed significant differences for the LTP-like stimulation conditions employing sinusoidal gratings, but not for the bar-like stimuli for either LTP- or LTD-like stimulation (“SG aligned LTP,” p = 0.0021; “SG misaligned LTP,” p = 0.045; “bar LTP”, p = 0.073; “bar LTD” experiment, p = 0.31). To get an estimate of these effect sizes, we calculated bootstrapped 95% confidence intervals (Fig. 4). These simulations yielded a confidence interval of change in JND of −0.25° to −0.54° for the “SG-aligned LTP” experiment. For the “SG misaligned LTP” and “bar LTP” condition, an intermediate effect of −0.18° to −0.42° and of −0.16° to −0.39° was found, respectively. This effect size was situated between that of the “SG-aligned LTP” experiment and the effect size in the sham experiment of + 0.00° and −0.34°. In the “bar LTD” experiment, this confidence interval encompassed the range between −0.30° and + 0.10°.

### Time course of improvement

**Figure 5:**
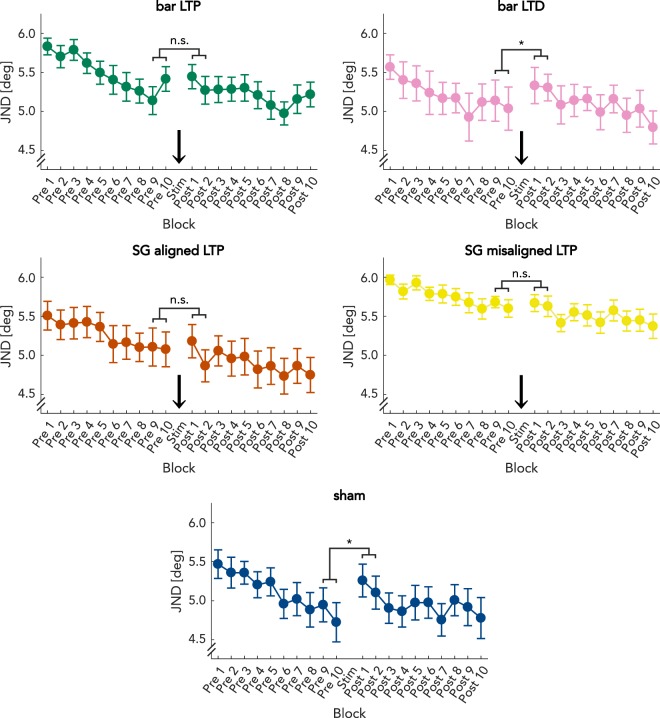
Blockwise representation of results. Mean JNDs for the 10 pre-session and 10 post-session blocks per group are shown. The time point of LTP-, sham-, or LTD-like stimulation is indicated by an arrow. Green: bar LTP. Purple: bar LTD. Red: SG aligned LTP. Yellow: SG misaligned LTP. Blue: sham. Error bars indicate standard error of the mean.

To gain further insight into possible mechanisms underlying the observed effects, we analyzed the time course of the perceptual changes (Fig. 5). This blockwise analysis showed that after LTP-like stimulation, the subjects maintained the performance level they showed at the end of the pre-session, while the subjects in the control experiments fell back to their initial performance shown at the very first blocks. To test this observation, we compared the means of the last two blocks of the pre-session to those of the first two blocks of the post-session for each experiment. For the “bar LTP,” experiment we found a nonsignificant increase in JND from 5.27° ± 0.16° to 5.35° ± 0.16° (ΔJND =  + 1.6%; Z = −1.39; p = 0.166). In the “bar LTD” experiment, there was a decrease in performance from 5.07° ± 0.26° JND to 5.32° ± 0.19° JND (ΔJND =  + 4.9%), which was significant (Z = −1.96; p = 0.049). In the “SG-aligned LTP” experiment, mean JNDs did not change significantly (pre: 5.06° ± 0.24°; post: 4.99° ± 0.21°; ΔJND = −1.3%; Z = 0.57; p = 0.568). Similarly, in the “SG-misaligned LTP” experiment, there was no significant change in the JNDs (pre: 5.69° ± 0.07°; post: 5.77° ± 0.08°; ΔJND =  + 1.3%; Z = −0.59; p = 0.557). The largest drop in performance was found in the sham experiment, where JNDs increased from 4.78° ± 0.23° to 5.14° ± 0.21° (ΔJND =  + 7.6%), (Z = −2.10; p = 0.036). These data suggest that LTP-like stimulation, in contrast to LTD-like or sham stimulation, prevents a loss of practice-related gain of orientation discrimination thresholds.

## Discussion

Our results show that 40 min of LTP-like visual stimulation with sinusoidal gratings are effective in altering perceptual performance in a fine orientation discrimination task whereas the LTD-like stimulation had no apparent effect. Surprisingly, this was also true in the “SG-misaligned LTP” experiment, in which the orientation feature used during stimulation was orthogonal to that of the behavioral assessment. Furthermore, LTP-like stimulation with an oblique bar stimulus that differed both in shape and size from the task stimulus had a weak, but not significant effect. It must be noted that in a sham control condition, there was a small but significant improvement of orientation discrimination ability. All modulatory effects of LTP-like stimulation were superimposed on top of any random difference observed in control conditions. This fact may explain our failure to find a significant interaction between session and stimulation type, hampering the interpretability of our data. For the LTD-like stimulation condition, we failed to elicit actual performance impairment and the small perceptual gain observed was not significant. Thus, our data suggest a modulatory influence of temporally patterned stimulation on orientation discrimination ability, with a possible gradient of efficacy from the most effective LTP-like stimulation protocol (SG-aligned LTP) over two intermediately effective protocols (SG-misaligned LTP, bar LTP) to the LTD-like stimulation protocol (bar LTD).

In our experiments with bar-like stimuli used during LTP-/LTD-like stimulation there are two differences with respect to the orientation discrimination task and the other stimulation protocols using sinusoidal gratings: Firstly, the bars differed in shape from the gratings used during orientation discrimination; Secondly, orientation was flickered between successive images^35^. Therefore, it remains unclear which of these two factors contributed to the small effect sizes in these conditions.

Classically, perceptual learning is thought to be specific with respect to stimulus features such as orientation^44^, spatial frequency^45^, retinal location^46^, motion direction^47^, background texture^48^, etc. Schoups, Vogels, & Orban^41^ demonstrated substantial feature specificity in learning during a fine orientation discrimination task, like the one employed in our experiments. However, recent research has shown that under special training conditions such as double training^49^ (however, see^50^) and short training^51^, specificity is reduced. Jeter *et al*.^51^ report that, even after several thousand trials, there is still some transfer to other features and locations. The number of baseline trials in our experiments was much smaller, which agrees with the partial transfer we observed. Furthermore, both top-down and passive bottom-up influences are sufficient for driving transfer in visual perceptual learning^52^. Despite the fact that the small effect sizes reported here make interpretation difficult, partial specificity may be explained in terms of a two-component model for perceptual learning^3^. According to this model, the interplay between feature-based and task-based plasticity may explain the differences in effect sizes between experimental conditions. There may be an interaction between the behavioral task and the subsequent stimulation which primes the stimulated location for both task-based and stimulus-based learning effects. The partial transfer we observed may reflect task-based learning, whereas the full effect only becomes manifest when congruent stimulus features during LTP/LTD-like stimulation also elicit feature-based learning. Investigating this matter would be difficult, as a straightforward approach to remove priming of learning from the task would need to have at least one experimental group without a baseline session.

The modest effect sizes in our experiments may in part be explained by results from experiments with task-irrelevant perceptual learning. Previous studies suggest that attentional processes actively inhibit suprathreshold task-irrelevant stimuli in favor of the relevant stimulus for the perceptual task^14,53^ and this has also been reported for orientation discrimination learning^54^. In the same vein, the effects of visual stimulation are known to be modulated by attention, with averted attention increasing transfer to other features and to tasks employing naturalistic images^55^. As far as our subjects were concerned, the “relevant” task during stimulation consisted of detecting slight movements of the fixation cross, while the flickered stimuli themselves were highly salient distractors, i.e., task-irrelevant for them. Therefore, attentional inhibition during stimulation may have weakened its effect. For LTD-like stimulation, this weakening may have been so strong that it masked the effect almost entirely. If a similar mechanism is at play, we could speculate that subthreshold stimulation would have a stronger effect in our current setup.

There is a substantial ongoing debate about possible sites of perceptual learning^3^. Electrophysiological and imaging studies report changes in low-level^42^ and intermediate visual areas^56–59^, along with higher-level cognitive areas^60,61^. Theoretical models have been developed that attempt to integrate these physiological as well as psychophysical findings. In one line of thought, these changes are interpreted in terms of changed readout of sensory representations by high-level decision-making areas, with only minor changes of sensory representations in low-level areas such as V1^62^. From another point of view, the cortical target of plastic changes is assumed to descend the cortical hierarchy with increasing task difficulty, leading to less transfer to other locations and stimulus features^63^. At different learning stages or under different task conditions, the current signal-to-noise bottleneck to further performance increases may differ^64^. Our results suggest a gradient of stimulation effectiveness that could affect several areas along the cortical hierarchy. However, only feature-specific stimulation may have reached the intermediate and lower-level areas, explaining its larger effect on discrimination performance. Even so, stimulation was applied at an early learning stage, with JNDs far from what has been reported after 15–20 days of training^41^. Feature-specific stimulation that affects early areas may be more effective in later stages of learning, when higher-precision judgments are needed and the internal noise in low-level sensory areas becomes the limiting factor.

Passive repetitive sensory stimulation approaches have been successfully introduced in the tactile domain^65^. In experiments using cutaneous stimulation, spatial summation was found to be necessary for cortical reorganization to occur^21^. Later experiments used LTP- and LTD-like low and high frequency stimulation, and demonstrated bidirectional changes of tactile discrimination abilities, within only 20 min^20^. Other studies employed electrical stimulation, which presumably activates all types of mechanoreceptive neurons, nonspecifically. Nonetheless, this spatially featureless stimulation enables functional reorganization of the somatosensory cortex through the recruitment of neighboring computational resources, resulting in enhanced tactile acuity, improved sensorimotor abilities in seniors^66^ and changes in pain perception^24^. In our experiments, we were able to utilize stimulation with specific stimulus feature characteristics that enabled us to investigate feature specificity in stimulation-based learning. We found an improvement in JND of approximately 7% for the most effective stimulation protocol. Ragert *et al*.^20^ found an improvement in two-point discrimination thresholds of 14%, twice as large in size. Furthermore, LTP-like stimulation of the fingertip only lasted 20 min. Tactile threshold improvements in other studies are of similar magnitude^21,65,67^. Threshold improvements after amphetamine application even reached a level of 23%^22^. Susceptibility to stimulation effects may thus be dependent on modality, and quite possibly on behavioral task as well.

In recent studies, it was demonstrated that there is a transfer of increased tactile acuity to other body locations^25,68^. After three hours of LTP-like electrical stimulation to the right index finger, tactile two-point discrimination thresholds were considerably lowered on the ipsilateral cheek and bilaterally at the lips. While these body regions lie far apart, their somatosensory representations lie close to each other at the cortical level, at the body/face discontinuity. It has been suggested that this spread of learning across the discontinuity is mediated by intracortical horizontal connections bridging the corresponding areas^69–71^. As horizontal connections in early visual cortex preferentially innervate neuronal populations with similar orientation preferences^72,73^, it is conceivable that an activation spread similar to that reported by Muret *et al*.^25,68^ is responsible for the comparatively stronger effect of our feature-congruent stimulation protocol.

Recent studies employing transcranial random noise stimulation (tRNS) have shown a facilitatory effect of high-frequency noise on visual perceptual learning in healthy and impaired populations^11–13^. Even though these interventions may appear similar at first sight, they differ in important respects. Frequencies used in tRNS span a higher range, than in our study and similar ones that investigate peripheral stimulation^20,35^. Furthermore, low frequencies are considered to lie in a range between 0.1 Hz and 100 Hz, whereas high frequencies go from 100 Hz up to 640 Hz^11^. The reasoning behind tRNS is to enhance the effect of temporal summation, bringing neurons in the affected system closer to their spiking threshold^74^. Furthermore, the random firing pattern is thought to prevent homeostatic adaptations in the affected neural populations that would bring them closer to their original resting activity^11^. In our experiment, LTP/LTD-like stimulation with relevant visual stimuli is hypothesized to induce high- or low-frequency activity in visual areas more directly, rather than accompanying perceptual learning. Nonetheless, both forms of stimulation appear to increase cortical excitability, which is in turn related to plastic changes^67,74^. It will remain to be seen how closely this interesting method is related to peripheral stimulation-based approaches.

The blockwise time course of learning between sessions showed a peculiar difference between LTP-like stimulation conditions on one side and sham and LTD-like stimulation conditions on the other side: Rather than plateauing, the subjects in the latter group reverted to a worse performance than that at the end of the pre-session. This is in accordance with previous findings, showing that a consolidating phase of sleep is necessary for the effects of perceptual learning to occur (e.g., see Fig. 9 in^41^). A sleep study on a similar orientation discrimination task reported a deterioration of performance without an intervening sleep period^75^ and consolidation is impaired in cases of disrupted sleep architecture^76^. Our data show that under conditions, in which subjects received visual LTP-like stimulation, performance in the post-session continued right where it left off at the end of the pre-session. Conceivably, two possible interpretations arise: The facilitatory effects of stimulation-based learning for orientation discrimination are similar in magnitude to those after a period of sleep, or more interestingly similar in mechanism. A possible method to investigate the latter would be to examine whether repetitive visual stimulation causes other sleep-like effects, such as the protection of learning from retroactive interference^77^.

Our results, despite modest effect sizes, show a clear gradient of visual stimulation efficacy depending on stimulus parameters and temporal pattern on orientation discrimination performance. In the study published by Schoups *et al*.^41^, the effect of training only became apparent after a period of sleep (their fig. 9). This finding makes it difficult to investigate the stability of the stimulation-induced learning in a follow up condition, as inter-day learning effects due to consolidation possibly mask stimulation-based performance changes. As in the case of LTD-like stimulation, a future study might remedy this by employing a task with a slow rate of learning between days or by deploying visual stimulation only when asymptotic performance has been reached.

Considering that we do not know the effect of visual stimulation on the shape of tuning curves in early visual areas, an important factor in the perceptual learning of orientation discrimination^42^, it is not clear whether stimulation oriented at the target orientation is the most effective. It may be the case that stimulation at an individual observer’s typically encountered orientations, or stimulation at a fixed offset from the reference orientation during the orientation discrimination task would be more effective. Adaptive stimulation protocols depending on a subject’s performance at baseline could address this limitation. A coarse orientation discrimination task under low contrast/low signal-to-noise conditions as behavioral assessment for perceptual learning could restrict the number of orientations to two constant orientations, also allowing for better controlled parametric testing of the relationship between stimulated and detected orientations, but would also arguably change the nature of the task toward a detection task.

## Material and Methods

### Participants

In total, we recruited 114 subjects (58 women, mean age: 24.27 ± 0.25 years) naive to the perceptual task to participate in the experiments. Of these, 25 subjects (12 women, mean age: 23.29 ± 0.53 years) took part in the “bar LTP” experiment, due to time constraints only 16 subjects (8 women, mean age: 24.88 ± 0.68 years) in the “bar LTD” experiment, 25 subjects (13 women, mean age: 23.88 ± 0.42 years) in the “SG aligned” experiment, another 23 subjects (13 women, mean age: 25.22 ± 0.63 years) in the “SG misaligned” experiment and 25 subjects (12 women, mean age: 24.32 ± 0.51 years) in the “sham” experiment. The subjects neither had neurological disorders nor were they under medication affecting the central nervous system, and had normal or corrected-to-normal vision. The experimental protocol was approved by the local ethics committee of the Ruhr-University Bochum, and was performed in accordance with the Declaration of Helsinki. All subjects provided written informed consent prior to participation. Seven subjects, not counted among the 114 subjects mentioned above, were excluded because their mean performance in the orientation discrimination task was at a chance level in at least one of the sessions (exclusions: bar LTP: n = 2; bar LTD: n = 0; SG aligned: n = 2; SG misaligned: n = 2; sham: n = 1). Due to the exploratory nature of these experiments, we did not perform power analyses beforehand.

### Apparatus and Stimuli

All stimuli were presented on an Iiyama HA202DT Vision Master™ Pro 512 22″ CRT monitor (K.K. Iiyama, Nagano, Japan) with a resolution of 1024 × 768 pixels, at a refresh rate of 100 Hz. Subjects sat in front of the screen at a distance of 65 cm with their heads on a chin rest. The surrounding room was dimly-lit (2–3 cd/m²). The experiments were conducted on a system running Windows XP.

#### Orientation Discrimination Task

We tested orientation discrimination performance using a custom program written in Java. For the assessment of orientation discrimination thresholds, the subjects had to indicate whether the orientation of the dark and light bars of a circular noise field (Fig. 1) deviated either clockwise or counter-clockwise from a virtual oblique target orientation, which was never presented^41^. The noise field was presented centrally on the screen and subtended 2.6° of visual angle. The bright parts of the grating consisted of bright and dark pixels randomly distributed at a ratio of 1:1. The dark parts consisted of uniformly distributed black pixels. The bright parts had a luminance of 75 cd/m² and the luminance of the dark pixels was 1.8 cd/m². The average stimulus luminance was 25 cd/m² while the background had a uniform luminance of 1.8 cd/m². To avoid giving positional cues to the subjects, the size and position of the grating stripes were randomized for each trial. The stimuli were presented on the screen for 400 ms, and the subjects had to respond within the next 600 ms. After this period, the next trial started with the onset of the next stimulus. The subjects had to indicate the perceived stimulus orientation by pressing a button on the keyboard (left and right arrow keys). Auditory feedback was given whenever a participant made a wrong decision or did not respond within the allotted time, which was also registered as a wrong answer. The initial orientation deviation was 7° in either direction which was reduced by 20% for four subsequent correct trials and was equally increased after each incorrect trial. One block consisted of 100 trials and JND was calculated as the logarithmic mean of reversal points of the staircase procedure, converging on an 84.09% correct response criterion^78^. For the session-wise JND, we calculated the geometric mean of 10 blocks.

### Repetitive LTP-/LTD-like Visual Stimulation

LTP-/LTD-like visual stimulation was applied by a custom program written in the Presentation® scripting language (Version 17.0, NeuroBehavioral Systems, Inc., Albany, California, USA). Three different stimulation protocols were tested that differed in timing and spatial configuration and orientation.

In the first type of stimulation, a bar stimulus was presented in an LTP-like fashion (Fig. 2C, “bar LTP”). One stimulation cycle consisted of a 5-s phase, during which stimuli flickered between the left and right oblique orientations, every 50 ms (20 Hz intra-train frequency), with a fixation cross on top of the image. In the latter 5-s phase of a cycle, only the fixation cross was presented on the background. This stimulation cycle was repeated 256 times, which took about 42 min. The bar stimulus was presented in the center of the screen with a luminance of 45 cd/m² and a length/width of 3.7° × 0.6°. During stimulation trains, this bar stimulus flickered between the right and left oblique orientations.

In the second stimulation protocol (Fig. 2C, “bar LTD”), spatial features were the same as above, but the stimulus was presented in an LTD-like manner. During LTD-like stimulation, the stimulus changed orientation at a frequency of 1 Hz and was presented continuously on the screen.

In a third stimulation protocol (Fig. 2C, “SG LTP”), the subjects were exposed to LTP-like stimulation with a SG instead of a bar stimulus. The temporal pattern was identical to that of the first LTP-like stimulation protocol. A SG with diameter of 3.4° and a λ of 1° was presented at the right oblique orientation. Peak luminance within these gratings was 100 cd/m² and lowest luminance was 1.8 cd/m², with a Michelson contrast of 96%. During stimulation trains, these gratings changed phase by 180°. Between stimulation cycles, the grating phase was randomly varied in four steps, each 90° apart.

In the sham experiment, only the fixation cross was presented on the gray background image. In the center of the screen, a small fixation cross was presented, which subjects were instructed to fixate at all times. To ensure that subjects kept their gaze locked onto the visual stimulation, infrequent catch trials had to be included, where the fixation cross would perform a small rightward movement of 1.1° from its central position. These catch trials occurred 32 times at random throughout the stimulation period. If a subject missed more than 8 catch trials, they were excluded from the subsequent analysis.

### Procedure

The subjects first performed 10 blocks of the orientation discrimination task (pre-session), with 1-min break between two subsequent blocks. Before the first block, the subjects received instructions and were familiarized with the task by conducting one block with assistance, which was later discarded from the results. All subjects except those in the “SG-misaligned LTP” experiment performed the task with the right oblique orientation as their reference orientation. The subjects in the “SG-misaligned LTP” experiment performed it with the left oblique as their reference instead. Thereafter, the subjects were exposed to either one of the three visual stimulation protocols or to the sham protocol, depending on the experiment they were assigned to (Fig. 2B). Stimulation was split into 8 blocks of equal length. After stimulation, the subjects had a break of 90 min in order to allow for consolidation processes to take place^35^ during which they were free to continue their daily activities. After the break, the subjects conducted the second set of 10 blocks of the orientation discrimination task (post-session).

Though a form of task-irrelevant perceptual learning (TIPL)^14^ in the wider sense, we want to conceptually separate our approach from this form of visual perceptual learning. In TIPL, subliminal stimuli that are irrelevant to a current task at hand are presented and usually coupled with a reward signal, whereas in stimulation-based learning suprathreshold stimuli are presented in a statistically optimized way that is thought to facilitate neural plasticity directly, without another task or means of reinforcement necessary.

### Statistical analysis

Statistical analyses were conducted by using MATLAB 9.0.0.341360 (The Mathworks Inc., Nattick, Massachusetts, USA) and the Statistics and Machine Learning toolbox (Version 10.2). Unless otherwise noted, results are given as mean value and standard error of the mean. JNDs were not normally distributed (Shapiro-Francia test of pooled baseline data: W = 0.83; p < 0.001) and variances were inhomogeneous (Brown-Forsythe test of pooled baseline data: F_4,109_ = 3.17; p = 0.017). Therefore, we calculated a permutated mixed-model ANOVA with a within-subject factor of session and a between-subjects factor of stimulation type to analyze the effect of visual stimulation on orientation discrimination ability. For the calculation of F-values after resampling, we randomly swapped individual pre- and post-session JNDs and performed a random permutation of group labels for all subjects. Randomized resampling was performed n_p_ = 10,000 times. The bias-corrected^79^ p-values were then calculated as the fraction of corresponding F-values that exceeded the empirical F-value from the original sample:

1 $$p=p({F}_{p} > {F}_{orig})$$

We conducted Wilcoxon signed-ranks as post-hoc tests to assess the statistical significance of pre-post changes. JNDs of single blocks, used during time course analysis, were also not normally-distributed, in general (not shown). Effect sizes were calculated with respect to the mean gain in JND of the sham condition (the standardized mean difference [SMD]) as.

2 $$SM{D}_{i}=\frac{{\bar{x}}_{i}-{\bar{x}}_{sham}}{\sqrt{{s}_{i}^{2}+{s}_{sham}^{2}}}$$

for the i^th^ experiment.

We used a bootstrapped version of the two-tailed paired-samples t-test, in which we tested against the null-hypothesis H_0_ that the change between pre- and post-session was equal to the change in JND in the sham group (ΔJND = −0.16°). In order to do this, data from each of the three stimulated conditions were transformed to conform to H_0_ by subtracting the mean group change in JND from each post-session data point and then adding the mean change in JND from the sham group.

3 $${x}_{post,{H}_{0}}={x}_{post}-{\bar{x}}_{post}+{\bar{x}}_{sham}$$

For each experiment, pre-post data pairs equal to the number of participants in a given experiment were randomly sampled, repeated for n_b_ = 1,000,000 times and paired-sample t-tests were performed on each bootstrap sample. The respective t_b_ values were compared with the calculated t-value based on the empirical data of each experiment and the bootstrapped p_b_ value was calculated as the fraction of bootstrapped t-tests in which t_b_ was larger than the empirical t-value, i.e.:

4 $${p}_{b}=p({t}_{b} > t|{H}_{0})$$

For p_b_ < 0.05, H_0_ was rejected. Bootstrapped confidence intervals were calculated using the bias corrected and accelerated method^80^.
